# The quality of invasive breast cancer care for low reimbursement rate patients: A retrospective study

**DOI:** 10.1371/journal.pone.0184866

**Published:** 2017-09-14

**Authors:** Shaofei Su, Han Bao, Xinyu Wang, Zhiqiang Wang, Xi Li, Meiqi Zhang, Jiaying Wang, Hao Jiang, Wenji Wang, Siyang Qu, Meina Liu

**Affiliations:** 1 Department of Biostatistics, Public Health College, Harbin Medical University, Harbin, PR China; 2 Department of Biostatistics, Public Health College, Inner Mongolia Medical University, Hohhot, PR China; 3 School of Medicine, University of Queensland, Royal Brisbane & Women's Hospital, Brisbane, Queensland, Australia; Tata Memorial Centre, INDIA

## Abstract

Though evidence-based treatments have been recommended for breast cancer, underuse of the treatments was still observed. To certain extent, patients’ access to care, which can be enhanced by increasing the coverage of health insurance, could account for the current underuse in recommended care. This study aimed to examine the association between different proportions of reimbursement and quality of recommended breast cancer care, as well as length of hospital stay. In this retrospective study, 3669 patients diagnosed with invasive breast cancer between 1 June, 2011 and 30 June, 2013 were recruited. Seven quality indicators from preoperative diagnosis procedures to adjuvant therapy and one composite indicator were selected as dependent variables. Logistic regression and generalized linear models were used to explore the association between quality of care and length of hospital stay with different reimbursement rates. Compared with UEBMI (urban employment basic medical insurance), which represented high level reimbursement rate, patients with lower rates of reimbursement were less likely to receive core biopsy, HER-2 (human epidermal growth factor receptor-2) testing, BCS (breast conserving surgery), SLNB (sentinel lymph nodes biopsy), adjuvant therapy and hormonal treatment. No significant difference in preoperative length of hospital stay was observed among the three insurance schemes, however URBMI (urban resident basic medical insurance) insured patients stayed longer for total length of hospital stay. Significant disparities in utilization of evidence-based breast cancer care among patients with different proportions of reimbursement were observed. Patients with lower rate of reimbursement were less likely to receive recommended care. Our findings could provide important support for further healthcare reform and quality improvement in breast cancer care.

## Introduction

Multidisciplinary care is recommended for invasive breast cancer, which includes surgical treatment with either mastectomy or breast-conserving surgery, adjuvant chemotherapy, adjuvant radiotherapy, and hormone therapy, etc. Appropriate use of the evidence-based care could reduce the likelihood of cancer recurrence, increase survival, and improve the quality of life [1, 2]. However, underuse of these effective treatments is widespread [3, 4]. Patient and hospital characteristics such as age, race/ethnicity and type of hospital were found to be associated with the receipt of cancer care [5, 6]. Besides, patients’ access to care, which could be enhanced by health insurance coverage, could account in part for the underuse of evidence-based care [7].With the increasing attention paid to cancer care and its relation with health insurance, a body of literature has examined whether disparities in quality of care and clinical outcomes existed for patients with varying insurance types [8–10]. Previous studies in USA have demonstrated that patients with no insurance or Medicaid were most likely to be diagnosed at later stages of cancer and receive less than optimal clinical care [9, 11, 12]; In addition, uninsured or Medicaid patients experienced higher mortality and lower survival compared to private insurance patients [8, 13]. As for breast cancer, though some studies have found significant influence of insurance type on utilization of evidence-based care, most of the studies only evaluated limited aspects of care. The continuum from preoperative diagnosis procedures to adjuvant therapy was seldom investigated.

In most instances, medical security system comprised several insurance types which have different coverages and proportions of reimbursement. For instance, in order to build a medical security system covering all the residents, the government of China had put forward three health insurance schemes, including UEBMI (urban employment basic medical insurance), NCMS (new rural cooperative medical scheme), and URBMI (urban resident basic medical insurance) [14].The coverage of three health insurance schemes had been expanded to 95.7% [15]. Among the three insurance schemes, UEBMI covers only 19% of population, but has the highest reimbursement rate: 85–95% varying with the amount of medical expenses. Reimbursement rate of patients covered by NCMS and URBMI ranges from 50% to 65% according to the level of hospital in which patients seek treatment, and they together cover 78% of Chinese population including rural residents and students, children, unemployed, elderly, disabled living in urban communities [16]. Therefor it is of great significance to pay more attention to the quality of care for most of the patients having sub-optimal financial support.

This study aimed to explore the influence of lower insurance reimbursement rates on utilization of evidence-based breast cancer care from preoperative diagnosis procedures to adjuvant therapy, which could contribute to the evidence pool based on which relevant healthcare policies would be designed to improve effective access to care particularly for severe disease such as cancer. Meanwhile, preoperative and total length of hospital were compared to examine the hypothesis that under- and over-consumption of health resources may exist across different insurance reimbursement rates.

## Materials and methods

### Data sources

In this retrospective study, participants were identified as women aged 18 to 69 years with a primary discharge diagnosis of invasive breast cancer (identified by International classification of disease version 10 diagnosis codes: C50.902, C50.151, C50.251, C50.351, C50.451, C50.551) between 1 June, 2011 and 30 June, 2013 who received all or part of their first course treatment in treating hospitals. Male patients, patients admitted to hospital for cancer recurrence and patients having bilateral or distant metastasis cancer were excluded (n = 445). Besides, patients with missing pathological information on tumor size or missing health insurance status were also excluded (n = 134). Finally, a total of 3669 patients who met the inclusion criteria were recruited in the study. Data were collected from medical records of patients diagnosed with invasive breast cancer in nine tertiary hospitals, including three specialized tumor hospitals and six general hospitals. This research was approved by Institutional Research Board of Harbin Medical University. Informed consent was obtained from all cases, and data was accessed anonymously.

### Data collection

The process of data collection is similar to one of our previous studies [17]. Information on patient demographics, tumor characteristics, diagnosis and treatment of breast cancer as well as data elements essential for identifying eligible patients for use of each treatment were extracted from medical records. Before the data collection, operation manual of data abstraction was formulated and data abstractors were trained for 2 weeks by an oncology professional and the leading researcher of the study team. During the formal data collection period, the leading abstractor randomly sampled 10% of the reviewed records and the inter-rater agreement was up to 95%.

### Dependent variables of interest

Seven of the quality indicators which were previously developed by our research team were determined as dependent variables of interest in the study [18]. The quality indicators included preoperative core biopsy, SLNB (sentinel lymph nodes biopsy), BCS (breast conserving surgery), human epidermal growth factor receptor-2 (HER-2) testing before systemic therapy, receiving at least 4 cycles of adjuvant chemotherapy, adjuvant radiotherapy after mastectomy, and hormonal treatment (Table 1). These indicators were examined throughout breast cancer care, from preoperative diagnosis to postoperative adjuvant therapy. Each quality indicator was dichotomous and expressed as percentage. While the denominator of the percentage denoted patients who were eligible without contraindications for the treatment, the numerator denoted eligible patients who actually received the treatment. A composite indicator was constructed by All-or-None method which represents the percentage of patients for whom all above indicators were completed. Analysis of each dependent variable was restricted to patients who were eligible for that treatment.

**Table 1:** Definitions of quality indicators.

Quality indicators	Eligible patients
Preoperative core biopsy	Patients with invasive breast cancer who underwent surgery
HER-2 testing	Patients aged 18 or over, with invasive breast cancer who received systemic therapy
Sentinel lymph nodes biopsy	Breast cancer patients with tumor size less than 3cm and negative clinical examination of axillary lymph nodes
Breast conserving surgery	Patients with stage I-II breast cancer
Receiving at least 4 cycles of adjuvant chemotherapy	Breast cancer patients who were administrated adjuvant chemotherapy
Adjuvant radiotherapy after mastectomy	Breast cancer patients who have received mastectomy
	and have tumor ≥ 5 cm or number of positive lymph
	node ≥ 4 or a T4 lesion
Hormonal treatment	Breast cancer patients with positive ER or PR, tumor
	size ≥ 1cm or positive axillary lymph nodes, and was
	not taking tamoxifen prior to diagnosis

Additionally, preoperative and total length of hospital stay of the first hospitalization for surgical breast cancer care were also examined as secondary outcomes of interest.

### Type of insurance

Information on health insurance status was collected from either patient discharge abstract or the home page of medical records. If the information of insurance was missing in both sources, the status of insurance was considered as unknown. Insurance status was categorized as three groups: NCMS, URBMI, UEBMI. The reimbursement rate for NCMS accounted for 50%-65%, which was much lower than the rate of 85%-95% for UEBMI but close to the rate of 50% for URBMI. Self-paid was not included as one of the categories since this group was heterogeneous, including both uninsured and commercial insured patients who cannot be distinguished from each other.

### Confounding variables

Patient characteristics, including age at diagnosis, income level, comorbidity condition, cancer stage, histological grade, ER/PR status, and HER-2, were adjusted as confounding variables. Since information on patient income could not be gathered, as an alternative, area-level annual per capita income was extracted from regional economy and society developed statistical bulletin 2012; income level was classified into lower income (<24565 RMB) and higher income (≥24565 RMB) groups according to the national annual per capita income in 2012 [19]. The information of pathological stage of cancer was extracted from medical records. An oncologist was additionally invited to carefully review the pathological reports and classify the stage of cancer when this information was not recorded. Twenty percent of patients with originally known cancer stage were randomly selected as a validation sample to verify the consistency between the oncologist’s and original cancer staging results, and the agreement rate was 95%. Patients were categorized as not receiving HER-2 testing when status of HER-2 showed positive with 2 plus and no further confirmation of HER-2 was performed through FISH or IHC testing. Furthermore, hospital characteristics were also considered when examining the effects of health insurance on utilization of breast cancer care and length of hospital stay. The hospitals were all public-owned and classified into specialized tumor hospital and general hospital. This study restricted all the investigated hospitals to teaching grade Tertiary hospitals located in northern China. For each dependent variable, pool of confounding variables differed slightly and were selected as priority on the basis of clinical relevance with receipt of each treatment.

### Statistical analysis

Baseline characteristics across different insurance groups were compared with Chi-squared test or Kruskal—Wallis H test depending on the type of variable. Differences in utilization of each quality indicator for breast cancer care among three insurance groups were tested using univariate logistic regression models. To examine the independent association between type of health insurance and utilization of quality indicators, multivariate logistic regression models were used adjusting for age, income level, comorbid condition, type of hospital, and, when appropriate, tumor characteristics (cancer stage, histological grade, tumor size, HER-2 status, and ER/PR). Since length of hospital stay was not normally distributed, generalized linear models with a gamma distribution and log link function were used to examine the association between health insurance type and length of hospital stay (preoperative and total length of hospital stay, separately). All statistical analyses were performed with SAS version 9.1 (SAS Institute, Cary, NC). Statistical significance was accepted at a level of *P*≤0.05 and all statistical tests were two-sided.

## Results

### Patient demographic and clinical characteristics

Patient and hospital characteristics by insurance type are shown in Table 2. In total, 3669 patients diagnosed with invasive breast cancer were included in the study. Patients’ median age was 49 years and interquartile range was 13. Twenty percent of the patients had at least one coexisting disease. Almost 86% of the patients resided in areas with lower annual per capita income. 77% were diagnosed with stage I and II breast cancer and 36% of the study population were treated at specialized tumor hospitals.

**Table 2:** Patient and hospital characteristics by insurance type.

Characteristics	Overall (n = 3669)	NCMS (n = 1216)	URBMI (n = 181)	UEBMI (n = 2272)	*P*
Age at diagnosis^†^, n (%)					<0.0001
<40	442 (12.05)	186 (15.30)	18 (9.94)	238 (10.48)	
40–50	1414 (38.54)	526 (43.26)	62 (34.25)	826 (36.36)	
50–60	1271 (34.64)	382 (31.41)	64 (35.36)	825 (36.31)	
>60	542 (14.77)	122 (10.03)	37 (20.44)	383 (16.86)	
Number of comorbidities^†^, n (%)					<0.0001
0	2934 (79.97)	1021 (83.96)	137 (75.69)	1776 (78.17)	
1	565 (15.40)	161 (13.24)	33 (18.23)	371 (16.33)	
≥2	170 (4.63)	34 (2.80)	11 (6.08)	125 (5.50)	
Income level^‡^, n (%)					<0.0001
Lower-income	3135 (85.45)	1099 (90.38)	141(77.90)	1895 (83.41)	
Higher-income	534 (14.55)	117 (9.62)	40 (22.10)	377 (16.59)	
Pathological stage^†^, n (%)					<0.0001
I	1229 (33.50)	323 (26.56)	45 (24.86)	861(37.90)	
II	1610 (43.88)	556 (45.72)	92 (50.83)	962 (42.34)	
III	830 (22.62)	337 (27.71)	44 (24.31)	449 (19.76)	
Hospital type^‡^, n (%)					0.2679
Specialized tumor hospital	1320 (35.98)	427 (35.12)	57 (31.49)	836 (36.80)	
General hospital	2349 (64.02)	789 (64.88)	124 (68.51)	1436 (63.20)	

^†^Wilcoxon rank test performed.

^‡^Pearson Chi-squared test performed.

All the patient characteristics distributed differently among the three insurance groups (Table 2). Compared with UEBMI and URBMI insured patients, NCMS insured patients were younger, less likely to have coexisting disease, and had lower level of income. Moreover, patients with NCMS and URBMI were more likely to be diagnosed at advanced stage (stage III) than patients with UEBMI. As for the choice of treating hospital (specialized tumor hospital or general hospital), no significant trend was found by insurance types.

### Disparities in utilization of breast cancer care

Among eligible patients, rates of receipt of preoperative core biopsy, HER-2 testing, SLNB, and BCS were 44.37%, 81.88%, 24.71%, and 9.57%. With regards to adjuvant therapy, 85.56%, 52.70%, and 37.86% of the eligible patients received at least 4 cycles of adjuvant chemotherapy, postoperative adjuvant radiotherapy and hormonal therapy, respectively. The rate of composite indicator was 6.79%. Table 3 also shows the adherence to each quality indicator by insurance type. Compared with UEBMI, patients of NCMS had significantly lower adherence to preoperative core biopsy, BCS and composite indicator. Patients insured by NCMS and URBMI had much lower adherence to SLNB and HER-2 testing than patients with UEBMI. In addition, a substantially lower percentage of patients with NCMS than patients with UEBMI and URBMI received adequate adjuvant chemotherapy and hormonal treatment.

**Table 3:** Unadjusted adherence to quality indicators by insurance type (%).

Quality indicators	Overall	NCMS	URBMI	UEBMI	P*
Preoperative Core biopsy (No.eligible = 3669)	44.37	40.30^a^	43.65	46.61	0.0016
HER-2 testing (No.eligible = 3669)	81.88	79.52^a^	76.80^a^	83.54	0.0026
SLNB (No.eligible = 1287)	24.71	21.39^a^	11.86^a^	26.96	0.0077
BCS (No.eligible = 2582)	9.57	5.13^a^	7.20	11.89	<0.0001
Receiving at least four cycles of adjuvant chemotherapy (No.eligible = 3400)	85.56	82.41^a^	88.69^b^	87.02	0.0009
Adjuvant radiotherapy (No.eligible = 852)	52.70	47.99	55.56	55.99	0.0728
Hormonal treatment (No.eligible = 2237)	37.86	26.13^a^	37.04^b^	43.77	<0.0001
Composite indicator (No.eligible = 3669)	6.79	4.44^a^	7.18	8.01	0.0003

* P values are based on univariate logistic regression.

^a^ denotes *P*<0.05 for pairwise comparisons, UEBMI was the reference group.

^b^ denotes *P*<0.05 for comparisons between NCMS and URBMI, NCMS was the reference group.

**Fig 1:**
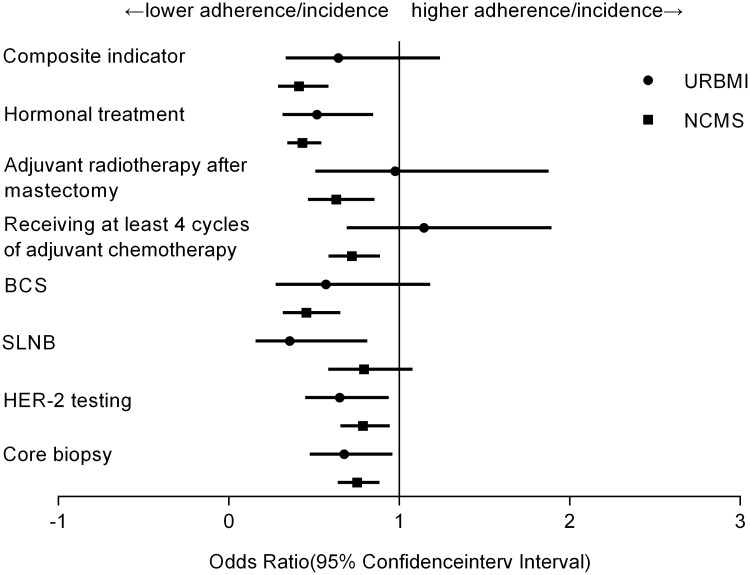
Adjusted association between type of insurance and utilization of breast cancer care. UEBMI is the reference group.

**Fig 2:**
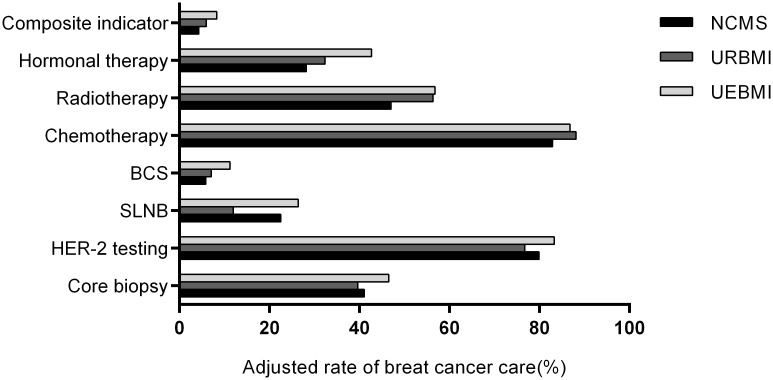
Estimated rates of breast cancer care by insurance type. *Chemotherapy* represents receiving at least 4 cycles of adjuvant chemotherapy; *Radiotherapy* represents adjuvant radiotherapy after mastectomy.

After adjusting for patient demographic and clinical characteristics as well as type of hospital (Fig 1), patients covered by NCMS and URBMI had lower odds of receiving preoperative core biopsy compared with UEBMI insured patients (for NCMS, OR = 0.752, 95%CI = 0.640–0.884; for URBMI, OR = 0.676, 95% CI = 0.475–0.961). No significant difference in utilization of SLNB was found between NCMS insured and UEBMI insured patients, but patients with URBMI were significantly less likely to receive SLNB than patients with UEBMI (OR = 0.357, 95% CI = 0.157–0.813). In the adjusted model, NCMS insured and URBMI insured patients were 21% and 35%, respectively, less likely than UEBMI insured patients to receive HER-2 testing. Disparities in the odds of receiving breast conserving surgery were also found across different insurance types: compared with UEBMI, NCMS insured patients had significantly lower odds of receiving breast conserving surgery (OR = 0.455, 95% CI = 0.317–0.655). In contrast with UEBMI insured patients, NCMS insured patients were 28% and 37% less likely to receive adequate adjuvant chemotherapy and postoperative radiotherapy than patients with UEBMI. Furthermore, NCMS and URBMI insured groups were both less likely to receive hormonal therapy compared to UEBMI group, OR = 0.432 and 0.517, respectively. NCMS insured groups also had lower rate of composite indicator (OR = 0.411, 95% CI = 0.289–0.585). The adjusted adherence to breast cancer care by insurance type is depicted in Fig 2.

Besides health insurance, other patient and hospital characteristics that had independent effect on the receipt of breast cancer care were found. Younger patients were more likely to receive standard care, except for adequate adjuvant chemotherapy and hormonal treatment. Higher income was positively associated with the receipt of SLNB, HER-2 testing, BCS, postoperative radiotherapy and hormonal treatment. Besides BCS, patients treated at specialized tumor hospitals were more likely to be provided with each aspect of breast cancer care. In addition, tumor characteristics such as cancer stage, tumor size, and histological grade also influenced the receipt of standard care (shown in S1 Table).

### Length of hospital stay

Comparisons of preoperative and total length of hospital stay among the three insurance groups are shown in Table 4. After adjusting for confounding variables, no significant difference in preoperative length of hospital stay was found among NCMS, URBMI, and UEBMI insured groups. However, patients with higher income or having received core biopsy had longer adjusted preoperative length of hospital stay. For total length of hospital stay, URBMI insured patients stayed longer in hospital when compared to patients with UEBMI (*P* = 0.0005). Besides, advanced cancer stage, receipt of core biopsy, and having mastectomy were associated with longer length of total hospitalization days.

**Table 4:** Preoperative and total length of hospital stay for breast cancer patients hospitalized for surgical care by type of insurance*.

Characteristics	Coefficient	SE	*χ*^2^	*P*
Preoperative length of hospital stay				
Insurance type (Reference: UEBMI)				
NCMS	-0.0202	0.0321	0.40	0.5295
URBMI	0.0331	0.0666	0.25	0.6200
Income level (Reference: Higher-income)	-0.1077	0.0406	7.03	0.0080
Core biopsy (Reference: Yes)	-0.2874	0.0328	76.9	< .0001
Total length of hospital stay				
Insurance type (Reference: UEBMI)				
NCMS	0.0054	0.0186	0.08	0.7715
URBMI	0.1317	0.0379	12.11	0.0005
Stage (Reference: III)				
I	-0.0587	0.0234	6.27	0.0123
II	-0.0184	0.0225	0.67	0.4127
Core biopsy (Reference: Yes)	-0.0422	0.0193	4.79	0.0286
Type of surgery (Reference: Mastectomy)	-0.1162	0.0314	13.72	0.0002

* Besides insurance type, only significant independent variables were showed (*P*<0.05).

## Discussion

In 2009, the State Council of the People’s Republic of China set realization of the universal basic health insurance coverage as one of the policy goals to be achieved during the new round of healthcare reform [20]. Although the universal basic health insurance coverage has been achieved by 2011, noticeable disparities existed among different reimbursement ratio.

The effect of insurance status on receipt of breast cancer care was reported by a number of studies [12, 21]. A study using data from the National Program of Cancer Registries found patients with Medicaid were less likely to receive guideline-concordant chemotherapy after adjusting for age, registry, and clinical variables [5]. Moreland A et al [22] observed that private insured patients were more likely to receive BCS than patients with Medicare or no insurance. However, few studies have explored the impact of health insurance on the continuum of breast cancer care from preoperative diagnosis procedures to postoperative adjuvant treatments. Moreover, to date, there were few studies that have examined the effect of health insurance on utilization of breast cancer care in China and other developing countries. Our study found substantial underuse in the continuum of breast cancer care and significant disparities in the receipt of treatments among different reimbursement ratio even after adjustment for patient and provider characteristics. Compared with UEBMI, patients with lower rate of reimbursement had less likelihood of receiving standard breast cancer care, including core biopsy, HER-2 testing, BCS and adjuvant treatment.

Preoperative core biopsy could inform patients of their diagnosis before the first treatment, thus enable them to make decisions about neoadjuvant chemotherapy and type of surgery [23]. The lower rate of core biopsy among patients of NCMS and URBMI may partly attribute to patient’ inactive involvement in the process of decision-making for treatment schedule. Receipt of core biopsy would prolong preoperative hospital stay, which was confirmed by our findings and other literatures [17]. Additional inpatient cost could be needed for the core biopsy. This may partially explain the lower rate of core biopsy among NCMS and URBMI insured patients, since those patients are very likely in worse economic condition and have less financial support. Confirmation of HER-2 status is critical for determining whether patients should receive biological target therapy [24]. In China, positive HER-2 status is presented in 20–30% of breast cancer patients. However, since biological target therapy is expensive and has not been covered by public health insurance during the study period, NCMS and URBMI insured patients, who had relatively low socioeconomic status, were discouraged to confirm the HER-2 status.

In the last decade, BCS has been recommended as preferred surgical modality for early-stage breast cancer worldwide. BCS could improve quality of life for patients with breast cancer and produce similar survival rate to mastectomy when it is combined with appropriate radiotherapy [25, 26]. In our study, the overall rate of BCS was only 9.57%, substantially lower than the rate in USA and other developed countries [27, 28]. Previous studies found characteristics of patients, including age, race, income, education, understanding of the disease and family support, were related to the choice of BCS [29–31]. In China, patients had misunderstanding of BCS and feared that BCS cannot completely resect the tumor, which, in the perception of most patients, will lead to recurrence and metastasis [32]. Moreover, radiotherapy, which is required after BCS and result in additional expenses, would also obstruct patients from receiving BCS. Doctors also played an important role in patient’s decision-making for surgical method. Several studies have observed that patients who received recommendations for BCS from doctors were more likely to receive BCS than those who were recommended for mastectomy [32, 33]. However, doctors were unlikely to recommend BCS to their patients, because of the strained relationship between doctors and patients in China [34]. To protect themselves from possible medical disputes, Chinese doctors were inclined to recommend mastectomy instead of BCS to patients. Our study found NCMS insured patients had the lowest rate of BCS relative to those insured by UEBMI, since patients insured by NCMS are rural populations who have relatively low income, poor understanding of disease, and less family support compared to UEBMI insured patients.

Receiving appropriate adjuvant therapy would reduce the likelihood of cancer recurrence and metastasis, and improve survival of breast cancer patients. Our study observed not only remarkable underuse of adjuvant treatments, but also the significant variations in utilization of those adjuvant treatments among patients with different insurance types. NCMS insured patients were unlikely to receive the adjuvant treatments, which could attribute to their low socioeconomic status, transport difficulties, and the lack of understanding of the importance of adjuvant therapy. A common problem with retrospective analysis is that data regarding use of endocrine therapy can be difficult to collect, since it consists of oral outpatient treatment. However, the patients taking hormonal therapy were identified based on information from three sources: discharge instruction, medication prescription record, and follow-up information in this study. Therefore, the oral outpatient treatment can be identified by any of the aforementioned means and the data about endocrine therapy was considered to be accurate enough. Still, we repeated the analysis excluding endocrine treatment, and the result did not differ substantially. The rate of composite indicator was 8.56%. Compared with UEBMI (9.77%), patients of NCMS (6.33%) had significantly lower score. After adjusting for confounding variables, NCMS insured group also had lower rate of composite indicator (OR = 0.500, 95% CI = 0.370–0.676).

Patient characteristics, including socioeconomic status, education background, and family/social support, tend to differ across types of health insurance. More important, the substantial variations in benefit coverage among the three health insurances schemes may also account for the disparities in utilization of breast cancer care. For example, the reimbursement rate of inpatients covered by NCMS and URBMI was 50%, which was much lower than that of UEBMI (85%-95%). Low reimbursement rate of medical expenses, which implies high out-of-pocket payment, could affect patients’ care-seeking behavior. To mitigate the financial risk caused by cancer care, as well as other major disease, and promote the utilization of care among the disadvantaged patients, China planned to set up complementary reinsurance program for major chronic illness among NCMS and URBMI insured patients [35]. Through the nationwide implementation of the reinsurance program and deepening of the healthcare reform, the disparities in utilization of breast cancer care across different insurance schemes could be gradually reduced.

To be noted, there were no significant differences in preoperative length of hospital stay across different insurance schemes, implying uniform preoperative waiting time regardless of insurance type. Beyond our expectation, the over- and under-consumption of health resources in UEBMI and NCMS insured patients were not observed. Patients insured by URBMI stayed longer in hospital than the other insurance groups, since they were older and more likely to present with coexisting diseases.

The study had several limitations. First, the impacts of health insurance on clinical outcomes of breast cancer patients were not examined; in the study, five-year survival of patient information was unavailable as the duration of follow up has not yet reached five years; meanwhile, inpatient mortality and occurrence of postoperative complications were both rare for patients with stage I-III breast cancer, which were inappropriate to be evaluated as short-term outcomes. Second, only tertiary teaching hospitals located in urban areas were included in the study, which may limit the generalization of our findings. In future studies, more types of hospital, such as non-teaching, private-owned, community and second-class hospitals, should be investigated, and potential interaction between hospital characteristics and health insurance on breast cancer care should be fully explored. Finally, due to the limitation of hospital information system, information of some confounding variables, such as education level and personal income, were not acquirable in the study. The area-level measure of income, instead of patient income, was gathered, which may attenuate the effect of income on utilization of care.

## Conclusions

The study observed substantial inequality of utilization of evidence-based breast cancer care across different insurance schemes. Compared with UEBMI, patients with NCMS and URBMI were less likely to receive preoperative diagnostic procedures, BCS, and adjuvant treatment, which suggested significant underuse of the continuum of breast cancer care among the insured patients with low level of reimbursement. The findings of this study provide important support for further health care reform aiming at equalizing the insurance benefits and the quality improvement in breast cancer care.

## Supporting information

S1 Table Adjusted association between type of insurance and utilization of breast cancer care. Only significant independent variables were showed, *P*<0.05.
(PDF)
