# Supplementary material for: The quality of invasive breast cancer care for low reimbursement rate patients: A retrospective study
Source: PLoS One. 2017 Sep 14;12(9):e0184866. doi: 10.1371/journal.pone.0184866 (PMC5599036; doi:10.1371/journal.pone.0184866)
Supplement: S1 Table — Only significant independent variables were showed, P<0.05. (PDF) [file pone.0184866.s001.pdf]

The quality of invasive breast cancer care for low reimbursement rate patients: a retrospective study.

Shaofei Su[1¶], Han Bao[2¶], Xinyu Wang[1], Zhiqiang Wang[3], Xi Li[1], Meiqi Zhang[1], Jiaying Wang[1], Hao Jiang[1], Wenji Wang[1], Siyang Qu[1], Meina Liu[1*].

[1] Department of Biostatistics, Public Health College, Harbin Medical University, Harbin, PR China.

[2] Department of Biostatistics, Public Health College, Inner Mongolia Medical University, Hohhot, PR China

[3] School of Medicine, University of Queensland, Royal Brisbane & Women's Hospital, Brisbane, Queensland, Australia

* Corresponding author

Email: liumeina369@163.com

¶These authors contributed equally to this work.

S1 Table. Adjusted association between type of insurance and utilization of breast cancer care*.

Quality indicators	Characteristics	OR	Lower 95% CI	Upper 95% CI
Core biopsy	Insurance type			
	NCMS	0.752	0.640	0.884
	URBMI	0.676	0.475	0.961
	UEBMI	Reference		
	Age at diagnosis			
	<40	1.656	1.222	2.244
	40-50	1.406	1.102	1.794
	50-60	1.315	1.033	1.672
	>60	Reference		
	Number of comorbidities			
	0	0.480	0.329	0.699
	1	0.694	0.463	1.040
	≥ 2	Reference		
	Income level			
	Lower-income	5.190	4.101	6.568
	Higher-income	Reference		
	Tumor size			
	≤2 cm	0.465	0.282	0.769
	2-5 cm	0.738	0.445	1.224
	>5 cm	Reference		

	Type of hospital			
	General hospital	0.151	0.127	0.180
	Specialized tumor hospital	Reference		
HER-2 testing	Insurance type			
	NCMS	0.787	0.655	0.945
	URBMI	0.650	0.449	0.939
	UEBMI	Reference		
	Age at diagnosis			
	<40	1.487	1.062	2.081
	40-50	1.559	1.200	2.025
	50-60	1.309	1.014	1.690
	>60	Reference		
	Income level			
	Lower-income	0.646	0.487	0.857
	Higher-income	Reference		
	Histological grade			
	Well differentiated	1.897	1.048	3.434
	Moderately differentiated	0.801	0.606	1.059
	Poorly differentiated	0.769	0.550	1.074
	Unknown	Reference		
	Type of hospital			
	General hospital	0.644	0.540	0.769

	Specialized tumor hospital	Reference		
SLNB	Insurance type			
	NCMS	0.793	0.583	1.079
	URBMI	0.357	0.157	0.813
	UEBMI	Reference		
	Age at diagnosis			
	<40	1.336	0.761	2.348
	40-50	1.661	1.057	2.610
	50-60	1.136	0.72	1.793
	>60	Reference		
	Income level			
	Lower-income	0.414	0.293	0.585
	Higher-income	Reference		
	Histological grade			
	Well differentiated	1.149	0.613	2.154
	Moderately differentiated	1.017	0.673	1.536
	Poorly differentiated	0.448	0.238	0.843
	unknown	Reference		
	Type of hospital			
	General hospital	0.496	0.370	0.665
	Specialized tumor hospital	Reference		
BCS	Insurance type			

	NCMS	0.455	0.317	0.655
	URBMI	0.570	0.275	1.182
	UEBMI	Reference		
	Age at diagnosis			
	<40	3.998	2.217	7.212
	40-50	2.286	1.347	3.879
	50-60	1.212	0.709	2.070
	>60	Reference		
	Income level			
	Lower-income	0.183	0.134	0.250
	Higher-income	Reference		
	Stage			
	I	2.189	1.642	2.918
	II	Reference		
Receiving at least 4 cycles of adjuvant chemotherapy	Insurance type			
	NCMS	0.721	0.586	0.887
	URBMI	1.145	0.693	1.894
	UEBMI	Reference		
	Stage			
	I	1.760	1.353	2.288
	II	1.435	1.137	1.812
	III	Reference		

	Histological grade			
	Well differentiated	2.023	1.171	3.494
	Moderately differentiated	2.099	1.605	2.747
	Poorly differentiated	1.679	1.198	2.351
	unknown	Reference		
	Type of hospital			
	General hospital	0.482	0.395	0.589
	Specialized tumor hospital	Reference		
Adjuvant radiotherapy after mastectomy	Insurance type			
	NCMS	0.631	0.465	0.855
	URBMI	0.976	0.508	1.876
	UEBMI	Reference		
	Age at diagnosis			
	<40	2.122	1.202	3.746
	40-50	1.802	1.136	2.860
	50-60	1.451	0.925	2.275
	>60	Reference		
	Income level			
	Lower-income	0.336	0.196	0.576
	Higher-income	Reference		
	Type of hospital			
	General hospital	0.321	0.238	0.431

	Specialized tumor hospital	Reference		
Hormonal treatment	Insurance type			
	NCMS	0.432	0.343	0.544
	URBMI	0.517	0.315	0.847
	UEBMI	Reference		
	Number of comorbidities			
	0	1.511	0.908	2.516
	1	1.977	1.148	3.405
	≥ 2	Reference		
	Income level			
	Lower-income	0.065	0.047	0.090
	Higher-income	Reference		
	Stage			
	I	0.637	0.487	0.832
	II	0.699	0.543	0.899
	III	Reference		
	Adjuvant chemotherapy			
	Yes	1.671	1.273	2.193
	No	Reference		
	Type of hospital			
	General hospital	0.418	0.337	0.517
	Specialized tumor hospital	Reference		

Composite indicator	Insurance type			
	NCMS	0.411	0.289	0.585
	URBMI	0.643	0.334	1.240
	UEBMI	Reference		
	Age at diagnosis			
	<40	2.102	1.182	3.736
	40-50	1.556	0.942	2.570
	50-60	1.254	0.765	2.055
	>60	Reference		
	Income level			
	Lower-income	0.135	0.098	0.184
	Higher-income	Reference		
	Stage			
	I	0.126	0.086	0.184
	II	0.125	0.088	0.178
	III	Reference		
	Type of hospital			
	General hospital	0.150	0.098	0.184
	Specialized tumor hospital	Reference		

*Only significant independent variables were showed, $P<0.05$.
